# Clinical outcomes of Tightrope system in the treatment of purely ligamentous Lisfranc injuries

**DOI:** 10.1186/s12893-021-01394-x

**Published:** 2021-11-07

**Authors:** Fan Yongfei, Liu Chaoyu, Xu Wenqiang, Ma Xiulin, Xu Jian, Wang Wei

**Affiliations:** Department of Orthopaedic Surgery, Anhui Spinal Deformity and Clinical Medical Research Center, Fuyang Hospital Affiliated to Anhui Medical University, Fuyang People’s Hospital, Fuyang, 236000 Anhui People’s Republic of China

**Keywords:** Tightrope system, Lisfranc ligament, Flexible fixation, Mid-foot function

## Abstract

**Background:**

Purely ligamentous Lisfranc injuries are mainly caused by low energy damage and often require surgical treatment. There are several operative techniques for rigid fixation to solve this problem clinically. This study evaluated the effect of using the Tightrope system to reconstruct the Lisfranc ligament for elastic fixation.

**Methods:**

We retrospectively analyzed 11 cases with purely ligamentous Lisfranc injuries treated with the Tightrope system from 2016 to 2019, including 8 male and 3 female. X-ray was performed regularly after operation to measure the distance between the first and second metatarsal joint and the visual analogue scale (VAS) score was used to evaluate pain relief. American orthopedic foot & ankle society (AOFAS) and Maryland foot score were recorded at the last follow-up.

**Results:**

The average follow-up time was 20.5 months (range, 17–24). There was statistically significant difference in the distance between the first and second metatarsal joint and VAS score at 3 months, 6 months, and the last follow-up when compared with preoperative values (*P* < 0.05).Mean of postoperative AOFAS mid-foot scale and Maryland foot score were 92.4 ± 4.3, 94.1 ± 3.5, respectively. The Tightrope system was not removed and the foot obtained better biomechanical stability. No complications occurred during the operation.

**Conclusion:**

Tightrope system in the treatment of purely ligamentous Lisfranc injuries can stabilize the tarsometatarsal joint and achieve satisfactory effect.

## Background

The tarsometatarsal joint is a multi-articular system composed of metatarsal bone, cuneiform bone, cuboid bone and the ligaments attached to them, which is essential for the function and stability of the mid-foot. There are three kinds of ligaments in the tarsometatarsal joint: dorsal ligament, interosseous ligament and plantar ligament, whereas there is no ligament connection between the first and second metatarsal bases. Lisfranc ligament is one of the thickest and most prominent interosseous ligaments. It originates from the lateral surface of the medial cuneiform inserted into the medial surface of the second metatarsal base, which is a stable structure connecting the medial column and the middle column [1–4].

Low energy tarsometatarsal joint damage is often associated with purely ligamentous Lisfranc injuries. It is difficult to diagnose after injury because the position of Lisfranc ligament is deep [2, 3]. Up to about 20–40% of ligamentous Lisfranc injuries are either mis-diagnosed or overlooked during initial evaluation [5]. Improper or untimely treatment can lead to the loss of bone stability of the transverse arch, diastasis between the medial and middle columns of the foot, and eventually mid-foot collapse, chronic pain and traumatic arthritis. Ligamentous Lisfranc injuries require extensive surgical intervention, including closed/open reduction with percutaneous puncture, open reduction and internal fixation, and primary arthrodesis. However, the tarsometatarsal joint is one of the amphiarthrosis and limitations of rigid fixation include iatrogenic articular cartilage injury, implant fracture and the need to remove implants [4, 6].

Recently, flexible fixation for the treatment of ligament injuries has been gradually recognized and popularized. The Tightrope system is a commonly used flexible internal device, which has been successfully used in the treatment of acromioclavicular joint dislocation and anterior cruciate ligament reconstruction. In the previous experiments, most of the patients returned to their sport/recreational athletic activities and their work smoothly [7–10]. This paper describes a technique of reconstructing Lisfranc ligament with the Tightrope system for the treatment of purely ligamentous Lisfranc injuries. The authors suggest that the use of this flexible structure provides better biomechanical stability, which not only duplicates the local anatomical structure, but also makes the mid-foot function return to the state close to its pre-injury earlier.

## Methods

**Fig. 1:**
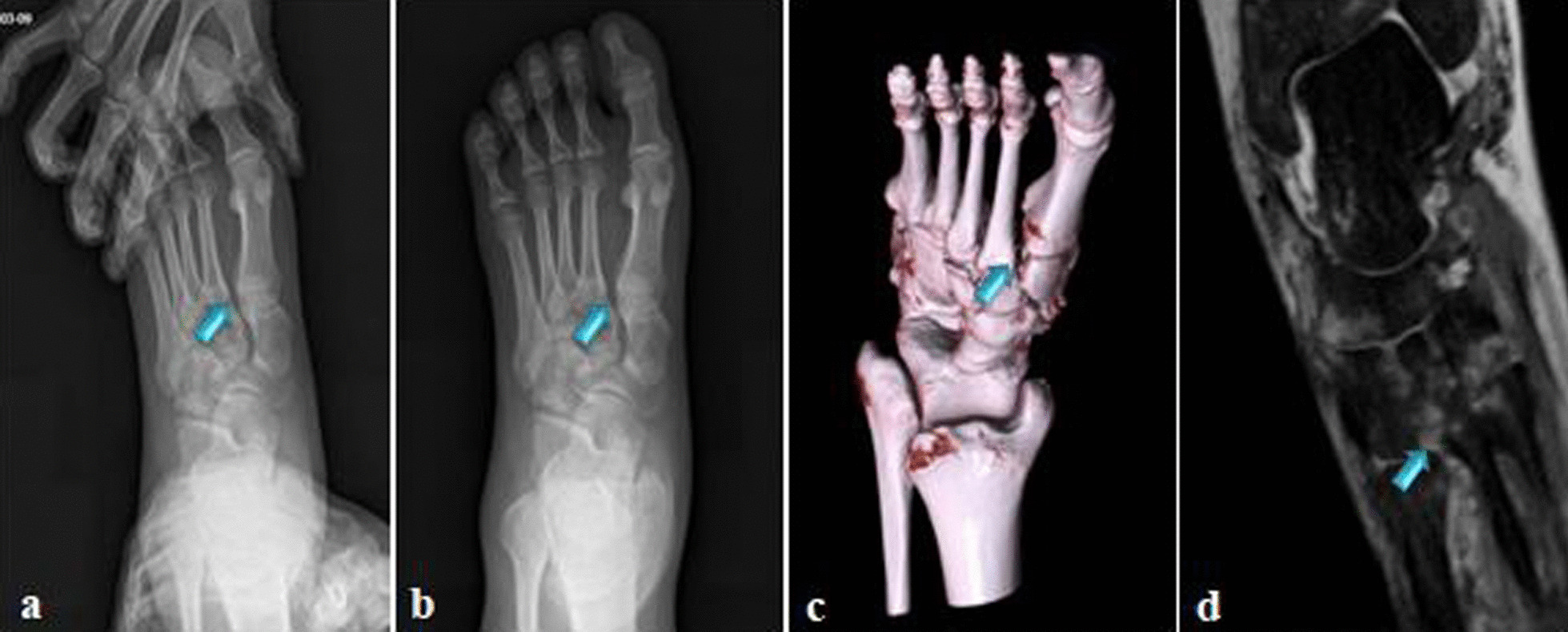
**a**–**d** Preoperative stress view, anteroposterior (AP) foot radiograph, weight-bearing CT and MRI of 27-year-old male patient with purely Lisfranc ligament injuries (blue arrows showing injury)

A retrospective study of 11 cases (8 male and 3 female) with a mean age of 35.4 (range 16–53) years who sustained purely ligamentous Lisfranc injuries from May 2016 to July 2019 was conducted. All cases were diagnosed as purely ligamentous Lisfranc injuries by medical history taking, traumatic mechanism, careful physical examination, non-weight bearing X-ray, stress views, weight-bearing CT and Magnetic resonance imaging (MRI) (Fig. 1). The exclusion criteria were patients with pathological fracture, old fracture, comminuted fracture of medial cuneiform bone and second metatarsal, open fracture with nerve, blood vessel and severe soft tissue injury, and loss to follow-up.

The traumatic mechanisms were mainly foot sprain in the mid-foot with a plantar flexion force, including falling down while running, jumping from a height and ball games. The typical physical examination findings included arch collapse, local pain and tarsometatarsal instability. The sensitivity of X-ray in the diagnosis of tarsometatarsal joint injury was weak, while weight-bearing CT could show a displacement of more than 2 mm between the first and second metatarsal joint, indicating avulsion of the Lisfranc ligament. In addition, MRI could further evaluate the injury degree of Lisfranc ligament. These imaging examinations can avoid the mis-diagnosis of ligamentous Lisfranc injuries. The Tightrope system was used to treat patients with purely ligamentous Lisfranc injuries and the distance between the first and second metatarsal joints was more than 2 mm in our study.

The mean interval from the initial injury to the operation was 4.5 (range 3–8) days (Table 1). After the operation, the limb was raised and a short-leg plaster splint was used to fix the ankle in 90° for 6–8 weeks. The foot was allowed to bear partial weight until the plaster splint was removed. By 3 months, the patients were able to walk with full weight and they had returned to almost full activity. X-ray was performed pre-operation, 3 months and 6 months after operation, and the last follow-up to measure the minimum distance between the first and the second metatarsal joint. Simultaneously, pain relief was evaluated by VAS score. Moreover, at the last follow-up, AOFAS score and Maryland score were used to evaluate the recovery of mid-foot function.

**Table 1:** Characteristics of patients

Patient	Sex	Age	Affected side	Traumatic mechanism	Time from injury to operation (days)
1	Male	27	Left	Falling down while running	4
2	Male	16	Left	Jumping from a height	6
3	Male	24	Left	Ball games	3
4	Male	29	Right	Ball games	3
5	Male	36	Left	Falling down while running	5
6	Male	31	Left	Ball games	4
7	Male	43	Right	Jumping from a height	4
8	Male	53	Left	Jumping from a height	8
9	Female	21	Right	Falling down while running	4
10	Female	27	Left	Jumping from a height	4
11	Female	36	Right	Falling down while running	5

### Ethics approval and consent to participate

All methods were carried out in accordance with relevant guidelines and provisions of the Helsinki declaration. The study protocol was approved by the Ethics Committee of Fuyang people’s Hospital (approval date/issue: 2019/17/3), and informed consent was obtained from the participants.

### Operative procedure

**Fig. 2:**
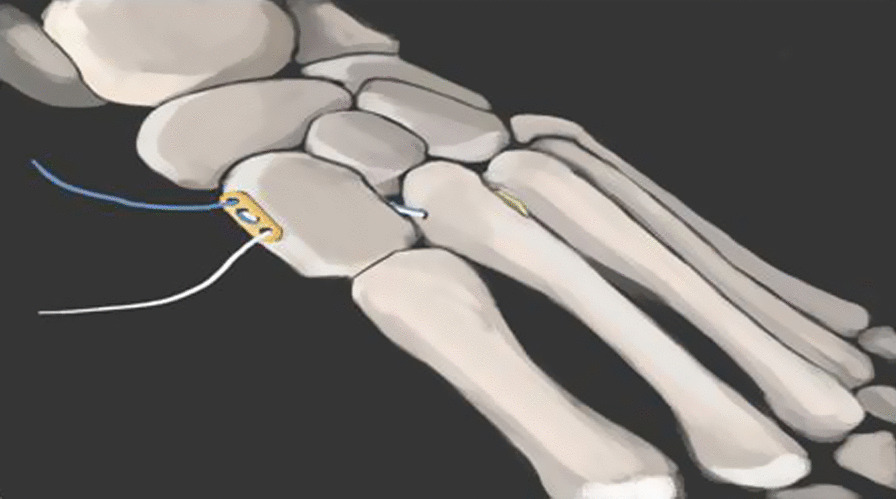
Diagram of Lisfranc ligament reconstruction with Tightrope system

**Fig. 3:**
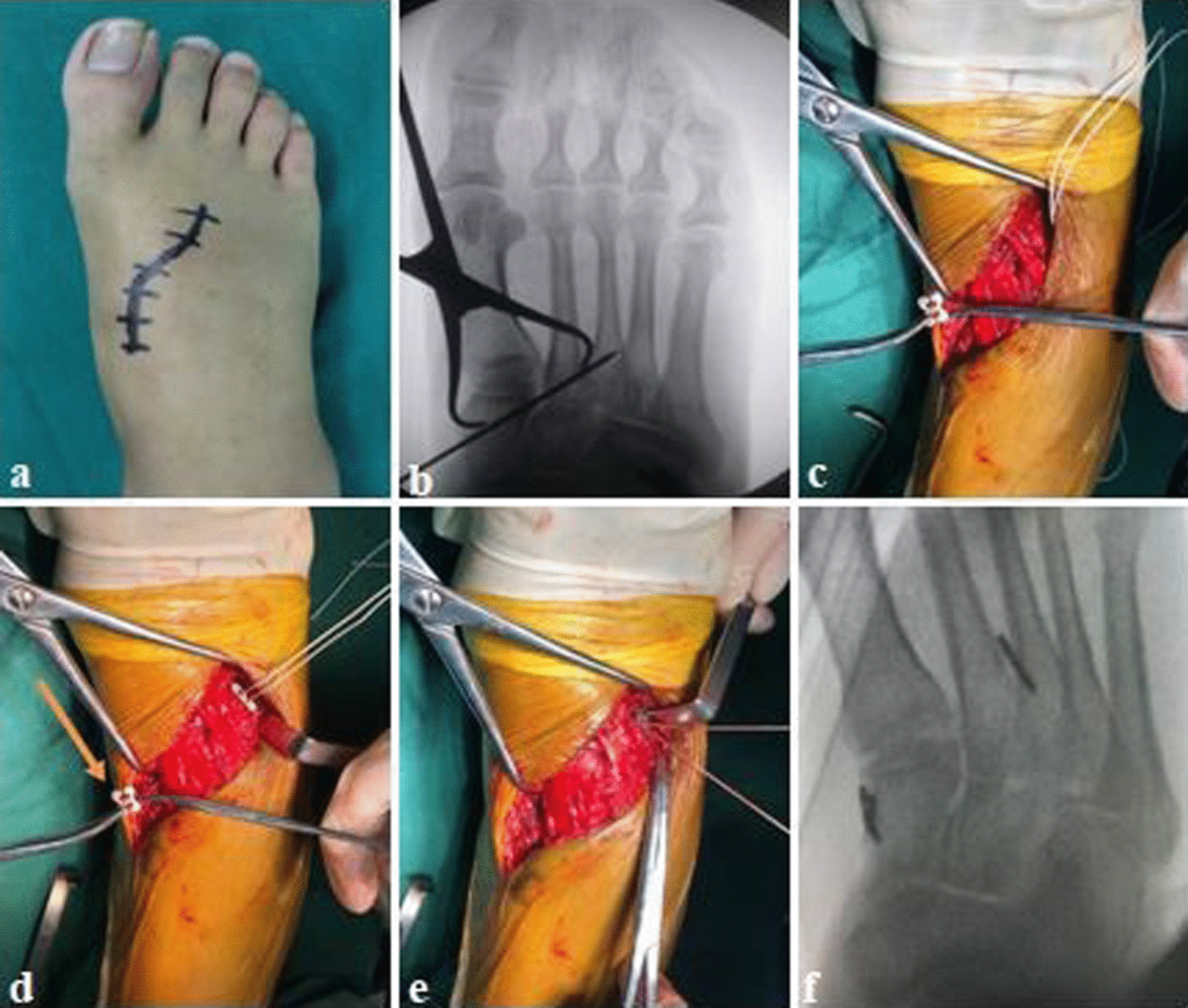
**a** A S-shaped incision was made between the first and second metatarsal; **b** fluoroscopy showed the reduced position and insertion of the K-wire; **c**–**e** Tightrope system was installed and tighten by high-strength suture (yellow arrow showing the button); **f** degree of reduction was observed under fluoroscopy

Under continuous epidural anesthesia with a tourniquet, a S-shaped incision was made between the first and second metatarsal to avoid the injury of adjacent neurovascular bundle. At the same time, the dorsal artery and tendon of foot should be protected. We exposed extreme instability of the tarsometatarsal joint and the ligamentous Lisfranc injuries. The soft tissue fragment was then removed from the tarsometatarsal joint in order to anatomically reset the base of the second metatarsal. Bone tenaculum was placed on the medial surface of the medial cuneiform bone and the lateral surface of the second metatarsal base to assist reduction. A 2.0-mm K-wire and a 3.5 mm hollow drill were inserted from the medial surface of the medial cuneiform bone to the second metatarsal base under fluoroscopic guidance to establish the ligament reconstruction tunnel, which was in line with the anatomical axis of the Lisfranc ligament. The Tightrope system (Arthrex Inc, Naples, FL, USA) composed of two buttons connected to each other by fiber wire. One button was installed on the lateral surface of the base of the second metatarsal bone, and the fiber wire was used to lead out to the medial of the medial surface of the medial cuneiform bone through the bony tunnel, and then the other button on the inside was tighten [11]. Until fluoroscopy confirmed that the position of internal fixation and reduction was satisfactory, the incision was washed and sutured layer (Figs. 2 and 3). At the end of the operation, the foot was held in a short-leg plaster splint with no weightbearing for 6–8 weeks.

### Statistical analysis

The SPSS 21.0 software (SPSS Inc., IL, USA) was used for data analysis. Quantitative data was expressed as means ± standard deviations (SD). The data in pre-operation and post-operation were compared by ANOVA. LSD test was used for pairwise comparison. *P* < 0.05 is considered as significant.

## Results

**Fig. 4:**
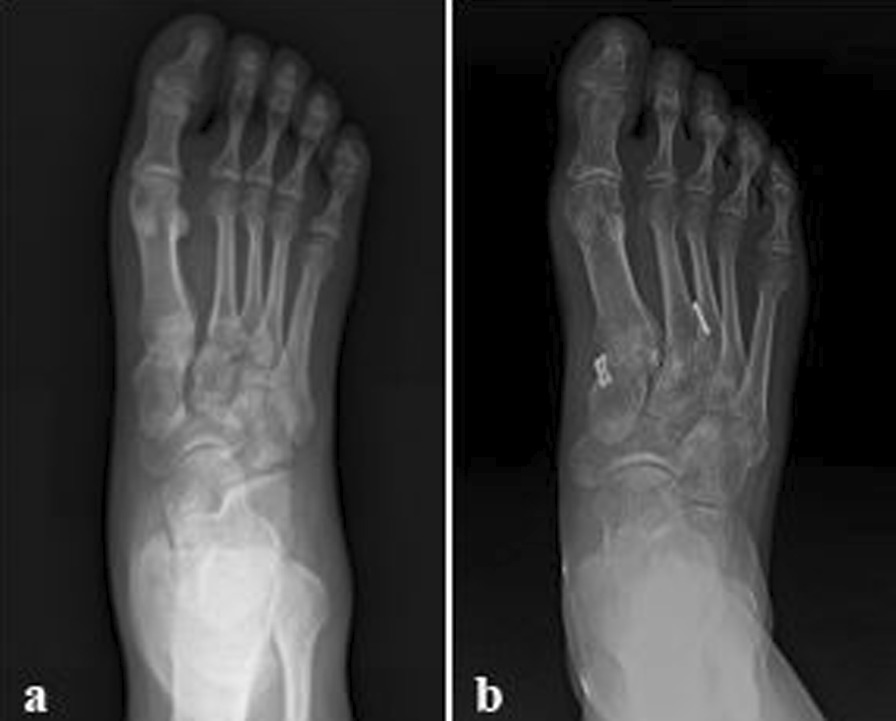
**a**, **b** Anteroposterior (AP) foot radiograph of early postoperative and the last follow up

The Tightrope system was not removed after operation and the average follow-up time was 20.5 months (range, 17–24). All the incisions healed in the first stage, and there were no immune rejection, incision infection in post-operation. Moreover, no screw loosening, plate fracture or other complications occurred during the follow-up time. At 3 months (1.7 ± 0.4), 6 months (1.8 ± 0.4) after the operation and the last follow-up (1.9 ± 0.5), the distance between the first and second metatarsal joint was significantly shorter than preoperative (8.9 ± 3.7) (*P* < 0.05) (Fig. 4). Similarly, VAS score decreased from 7.1 ± 1.0 in preoperative to 1.8 ± 0.6, 1.7 ± 0.9, 1.5 ± 0.7, respectively (*P* < 0.05). Nevertheless, there was no significant difference between the time points after operation (*P* > 0.05) (Table 2). These data showed that the foot obtained a better biomechanical stability, and there was no obvious reduction loss and the symptoms of foot had evidently been relieved. In the last follow-up, the mean AOFAS mid-foot scale and Maryland score were 92.4 ± 4.3 (range, 85–97) and 94.1 ± 3.5 (89–98), respectively. This outcome suggested that the foot function had been well recovered.

**Table 2:** Comparison of the distance between the first and the second metatarsal joint and VAS scores between pre- and post-operation ($\overline{x} \pm {\text{s}}$)

Time	Distance between the first and the second metatarsal (mm)	VAS score
Pre-operation	8.9 ± 3.7^#Δ^	7.1 ± 1.0^#Δ^
Three months after operation	1.7 ± 0.4*	1.8 ± 0.6*
Six months after operation	1.8 ± 0.4*	1.7 ± 0.9*
Last follow-up	1.9 ± 0.5*	1.5 ± 0.7*

^*^Compared with pre-operation value, *P* < 0.05

^#^Compared with the value at 3 months after operation, *P* < 0.05

^Δ^Compared with the value at 6 months after operation, P < 0.05

## Discussion

The metatarsal joint can be divided into three parts according to the anatomical structure characteristics, including the medial column, middle column and lateral column, which is an important structural component of the mid-foot. It is located between the metatarsal bone and tarsal bone, forming a unique “arch” structure in the cross section which maintains the lateral stability of the joint. The second metatarsal bone is inserted between the medial cuneiform and lateral bone to form a mortise and tenon structure, which becomes a “wedge stone” for the longitudinal stability of the tarsometatarsal joint [12, 13]. The incidence of tarsometatarsal joint injury accounts for 0.2% of all fractures, which is not related to gender [4]. The tarsometatarsal joint injury represents a series of injuries, ranging from high-energy damages, with severely unstable mid-foot, to low-energy damages, with subtle subluxations or instability without significant displacement. Low energy damages are the most common type, usually accompanied by ligamentous Lisfranc injuries, which are the result of axial, rotational or twisting injuries, especially in sports such as running, jumping and twisting the weightbearing foot [4, 14–16]. Lisfranc ligament is the most important kind of interosseous ligament, which is a stable structure connecting the medial column and the middle column, and it had a single bundle in 73% and double bundles in 27% of the feet [17]. Ligamentous Lisfranc injuries are difficult to be found by X-ray because of its unique anatomic location and natural morphology, which leads to the failure diagnosis [14]. It is the reason for adverse clinical outcomes that patients with purely ligamentous Lisfranc injuries get inappropriate treatment due to misdiagnosis or underestimation of the severity of the injury. In order to reduce mis-diagnosis and improve the treatment of patients, weight-bearing CT and MRI are used to detect the occult Ligamentous Lisfranc injuries and evaluate the foot recovery in this study.

We treated the patients with the tarsometatarsal joint injury in light of the degree of damage. A short-leg plaster splint was used to fix the foot with no weightbearing for 4–6 weeks for patients, with stable tarsometatarsal joint without separation or displacement less than 2 mm, which achieved a good therapeutic effect. This kind of conservative treatment is not suitable for patients whose displacement of tarsometatarsal joint is more than 2 mm and the surgery is particularly necessary in order to restore the biomechanical stability of the foot. If the ligamentous Lisfranc injuries is repaired only by fibrous regeneration and scar healing, whose strength will decrease and length will be elongated, which can lead to long-term complications such as chronic foot pain and limitation activity, and then seriously affect the foot function and quality of life in patients [4, 18].

The treatment of ligamentous Lisfranc injuries is always a controversial topic in clinics. Various operative strategies had been described such as closed/open reduction and percutaneous puncture with K-wires, open reduction and internal fixation with transarticular screws or plates, and primary arthrodesis [3, 4, 19, 20]. Each of them had its own defects. Firstly, a large amount of cartilage was removed in primary arthrodesis, so that the articular surface was almost completely destroyed [21]. Additionally, the loss of mobility of the first tarsometatarsal joint would lead to compensatory hypermobility and adjacent joint sclerosis [6]. Reinhardt et al. [22] in their investigation suggested that a 12% rate of adjacent joint arthritis in a group of 25 patients who were followed for 42 months after primary fusion. The stability of foot was insufficiency whether open reduction or closed reduction with K-wires, and there was a high risk of nonunion or redislocation of tarsometatarsal joint. Nowadays, open reduction and internal fixation with transarticular screws or plates had been the main choice for purely ligamentous Lisfranc injuries [2, 21, 23–25]. Traditional transarticular screws were a reliable rigid fixation method with good stability. Thus, patients with ligamentous Lisfranc injuries would walk off bed at an early stage after operation, which prevented the complications due to long-term in bed and reduced the risk of foot deformity. But there was still a great deal of drawbacks in this technique. On the one hand, the insertion of screws would increase the damage of articular cartilage, leading to the occurrence of secondary osteoarthritis. On the other hand, screws placement along the direction of the Lisfranc ligament would result in the disruption of the ligament attachments. A recent study had shown that the average injury areas to the Lisfranc ligament caused by the use of an intra-articular screw were 3.33 mm^2^ and 3.49 mm^2^, respectively, while the average percentage of damage at the medial cuneiform and the second metatarsal were 1.75% and 2.55% [21]. The increased destruction of Lisfranc ligament might interfere with the healing process and decreased the stability of the foot, which limited the foot activity. Plates could provide comparable biomechanical stability of the joint to screws. A novel dorsal plate had been developed which provided transverse as well as longitudinal stability and exerted a small impact on articular cartilage [2]. However, the additional trauma and the local instability were extended because of the internal fixation must be removed in both methods at the later stage.

In recent years, flexible fixation for the treatment of ligament injuries has gradually been mentioned. There was an investigation demonstrated that Lisfranc ligaments reconstruction using autologous graft provided comparable intensity of the fixation compared to the transarticular screw method, while conforming to the original anatomical structure [26, 27]. Miyamoto et al. [26] performed a technique that Lisfranc ligaments reconstruction using autologous gracilis tendon for five athletes who suffered from ligamentous Lisfranc injuries. Post-operative X-ray examination showed that the loss of joint reduction and the foot limitation activity were finite. Nevertheless, there still have a lot of problems to be explored in this technology as a result of the short application time, including procedure of tendon graft fixation, diameter of bone tunnel, selection of tendon graft and structural characteristics of tendon. The Tightrope system is one of the flexible internal fixations in clinic, which composed of two buttons connected to each other by fiber wire. The fiber wire is made of polyester suture around the ultra-high molecular polyethylene inner core, with reliable strength. The button is composed of stainless steel and titanium metal, with good biocompatibility [28–30]. Biomechanical studies showed that the fractures and tendon ruptures can lead to changes in the sagittal balance line for foot loading and pace training [31, 32]. The Tightrope system could potentially bring a more physiological fixation, reduce the foot loading and attain a dynamic stability, and avoid the incidence of traumatic arthritis, joint stiffness and other complications caused by rigid fixation for a long time. The Tightrope system was not removed after operation and the rate of instability and redislocation of joint was obviously decreased. Along the direction of Lisfranc ligament (the angle with the second metatarsal shaft is about 42° in sagittal plane and 15° in horizontal plane), a bone tunnel was designed between the medial cuneiform bone and the second metatarsal bone to reconstruct Lisfranc ligament by Tightrope system in the study. During the follow-up time, the distance between the first and second metatarsal bone was evidently shortened, the local pain was significantly relieved, the height of the foot arch was effectively maintained, and there was no obvious loss of joint reduction. At the last follow-up, the mean of postoperative AOFAS mid-foot scale and Maryland foot score were 92.4 ± 4.3, 94.1 ± 3.5, respectively. The mid-foot function returned to the state close to its pre-injury. We summarized the mentions for Lisfranc ligament reconstruction with Tightrope system. Firstly, the alteration of bone tunnel between the medial cuneiform bone and the second metatarsal bone should be prevented to avoid formation of “N” wrinkle of the implanted suture and reduce the friction between the suture and the bone tunnel during the foot movement, which eventually averted the disruption of the suture. Secondly, the knot should not be too large when knotting in the medial surface of the medial cuneiform bone; otherwise it would easily lead to subcutaneous foreign body reaction and inflammation. Finally, the active and passive motion should not be carried out too early.

## Conclusion

The reconstruction of Lisfranc ligament with Tightrope system can better stabilize the tarsometatarsal joint and obtain satisfactory foot function for patients with purely ligamentous Lisfranc injuries. However, there are only a few cases in this study and the follow-up time is short. Further investigation is needed to evaluate the long-term effect and compare the discrepancy with other ligament reconstruction methods.

## Data Availability

The datasets used and/or analyzed during the current study are available from the corresponding author on reasonable request.

## Abbreviations

VAS: Visual analogue scale
AOFAS: American orthopedic foot & ankle society
3D: Three dimension
MRI: Magnetic resonance imaging
